# Incremental Prognostic Value of Anemia in Acute Coronary Syndrome from A Rural Hospital in India

**DOI:** 10.5334/gh.527

**Published:** 2020-02-12

**Authors:** Anjalee Chiwhane, Shreerang Burchundi, Gajendra Manakshe, Hemant Kulkarni

**Affiliations:** 1 Department of Medicine, Jawaharlal Nehru Medical College, Sawangi, Wardha, IN; 2 Lata Medical Research Foundation, Nagpur, IN; 3 M&H Research, LLC, San Antonio, TX, US

**Keywords:** acute coronary syndrome, outcomes, epidemiology, anemia

## Abstract

**Background:**

Anemia is highly prevalent in low- and middle-income countries, where prevalence of acute coronary syndrome (ACS) is also rising. Evidence indicates that baseline anemia status can prognosticate ACS. However, the Global Registry of Acute Coronary Events (GRACE) score that is popularly used all over the world does not include information on anemia.

**Objectives:**

Our objective was to investigate if anemia at admission, along with the GRACE score, improves the prediction of adverse outcomes within 6 months in rural Indian patients of ACS.

**Methods:**

We enrolled 200 ACS patients at the Acharya Vinoba Bhave Rural Hospital—a rural, tertiary care hospital in central India. Patients were followed for 6 months for death and major adverse cardiac event (MACE). Improvement in the prediction of adverse events by including anemia in addition to the GRACE score was quantified using area under the receiver operating characteristic curve (AUC), integrated discrimination improvement (IDI) and the net reclassification index (NRI).

**Results:**

There were 31 deaths due to MACE and an additional 28 non-fatal MACE events during follow-up. Baseline hemoglobin was strongly and independently associated with both outcomes even after adjusting for a multivariable propensity score. For the outcome of death and death/MACE there was a moderate improvement in the AUC of 1% and 6%, respectively. However, for these outcomes the IDI for baseline hemoglobin was 6% (p = 0.03) and 12% (p << 0.0001), respectively, while the NRI was 0.50 (p = 0.01) and 0.78 (p << 0.0001), respectively.

**Conclusions:**

Inclusion of baseline anemia in addition to the GRACE score improves prognostication of ACS patients.

## Introduction

Acute coronary syndrome (ACS) represents a grim challenge to global cardiovascular health. An estimated 129 million disability-adjusted life years (DALYs) and seven million deaths are annually attributed to ischemic heart disease globally [1,2]. Nearly two-thirds of the DALYs and half of the IHD-related deaths occur in low- and middle-income countries (LMIC) [3]. In this context, it is noteworthy that low socio-economic status has been identified as a significant risk factor for occurrence and consequences of ACS in countries like India [3,4,5].

The significant differences between the ACS profiles in high income countries versus LMICs can be explained by the differences in the comorbidity pattern. Roy et al. [6] have demonstrated that the comorbidity patterns in countries with high versus low Human Development Index are vastly different. Emerging data from Indian studies show that the presence of anemia is associated with adverse outcomes after ACS [7,8]. Considering the high prevalence of anemia in India, especially in the rural populations, it is conceivable that the presence of anemia may contribute to ACS-related morbidity and mortality in these settings.

There is a strong biological rationale for anemia as a prognostic predictor in ACS patients. Reduced ability to carry oxygen to an already under-perfused myocardium [9], impaired vascular healing [10], increased inflammatory influx [11], heightened risk of thrombosis [12], need for whole blood or packed cell transfusions [13], and differing medication profiles [14,15] can all contribute to adverse outcomes in ACS patients with anemia. Considering these mechanistic explanations, anemia can be expected to influence both short-term and long-term outcomes after ACS [16,17,18]. In an elegant meta-analysis of 19 published studies covering data on 241,293 ACS patients, Liu et al. [19] concluded that anemia is an independent predictor of adverse outcomes and should be used for risk-stratification in ACS patients.

A popular method for risk stratification of ACS is the Global Registry of Acute Coronary Events (GRACE) risk score [20] which is based on data from approximately 250 hospitals representing 30 countries (http://www.outcomes-umassmed.org/GRACE/). It uses the following nine predictors: age, development (or history) of heart failure, peripheral vascular disease, systolic blood pressure, Killip class, initial serum creatinine concentration, elevated initial cardiac markers, cardiac arrest on admission, and ST segment deviation. This score does not include anemia as a possible independent and additive prognosticator. In this study, we tested the following hypotheses in a rural, tertiary care hospital in India: a) baseline hemoglobin concentration is an independent risk factor for adverse outcomes in ACS patients; and b) baseline anemia in addition to the GRACE score will improve the overall prognostic performance to predict adverse outcomes within six months of hospital admission.

## Methods

### Study population

This was a cross-sectional study with a six-month long follow-up for adverse outcomes after ACS. All consecutive patients of ACS who were admitted between 1st November 2014 to 31st December 2015 to the cardiovascular services of the Acharya Vinoba Bhave Rural Hospital, Sawangi, Wardha, India were included in this study. The study center is a teaching, multispecialty, 1,390-bed tertiary care hospital located in rural central India. All the enrolled patients provided contact details for collecting the follow-up data at the end of one month and six months from the time of index hospital admission. All patients gave a written, informed consent for enrollment into the study. The study protocol was approved by the Central Ethics Committee for Human Research at the Jawaharlal Nehru Medical College, Sawangi, Wardha, India.

### Outcomes and predictors

This study focused on two primary outcomes: a) death during six months of index admission and b) death or the first major adverse cardiac event (MACE) during six months of index admission. A MACE was defined as one or more of the following events: post-infarction angina (PIA, International Classification of diseases ICD-10-CM Diagnosis code I23.7), heart failure (HF, ICD-10-CM Diagnosis code I50), ventricular tachycardia (ICD-10-CM Diagnosis code I47.2) and ventricular fibrillation (ICD-10-CM Diagnosis code I49.01). Death was confirmed by a death certificate when available or was informed by the next of kin.

The predictor variables included body mass index (weight in Kg/height in m^2^); waist/hip circumference ratio; tobacco, alcohol and smoking habits assessed during personal interview; Killip class; presence of important comorbidities like diabetes, hypertension, dyslipidemia, transient ischemic attack (TIA)/stroke; and concomitant cardiovascular disease. Hypertension was defined as history of anti-hypertensive drug use or a systolic blood pressure of ≥140 mmHg or a diastolic blood pressure of ≥90 mmHg. Dyslipidemia was defined as described by Chou et al. [21] Use of cardiovascular medications was also inquired into with a focus on the use of aspirin, β-blockers, statins, angiotensin receptor blockers, angiotensin converting enzyme (ACE) inhibitors and potassium-sparing diuretic spironolactone.

GRACE score was estimated using the Fox equations for death and death/myocardial infarction [20] as described by Anderson and FitzGerald [22]. Baseline hemoglobin concentration was measured using automatic blood analyzer (Beckman-Coulter, Miami, FL). Anemia was defined as a hemoglobin concentration <13.0 g/dl in men and <12.0 g/dl in women [23]. Additionally, baseline serum creatinine and serum albumin levels were measured using RX-imola auto-analyser (Randox Laboratories, Kearneysville, WV).

### Statistical methods

Descriptive statistics included mean and standard deviation (SD) for continuous variables and proportions for discrete variables. Association with the study outcomes was tested for statistical significance using Fisher’s exact test, Person’s chi-square test or analysis of variance (ANOVA). Multivariable association with the study outcomes was tested using logistic regression analyses. To test the robustness of the findings from the logistic regression analyses, we conducted propensity score analyses. Propensity score was determined as the predicted probability from sequential logistic regression models to ensure balance of the covariates across patients with and without baseline anemia. We used the package prop_sel (http://personalpages.manchester.ac.uk/staff/mark.lunt) for these analyses. Quintiles of the propensity score were then used as a covariate along with anemia to predict the study outcomes in a logistic regression framework. The optimum operating point (OOP) for the GRACE scores was determined as the point on the receiver operating characteristic curve (ROC) that was closest to the upper-left corner of the ROC plot. Incremental value of baseline hemoglobin concentration over GRACE score was determined by comparison of the area under the receiving operating characteristics curve using the DeLong, DeLong and Clarke-Pearson test [24]. We also report the bootstrap-based 95% confidence intervals for differences in the area under the ROC as recommended by Demler et al. [25] using the package comproc [26]. Lastly, we estimated the incremental discrimination improvement (IDI) and the continuous version of the net reclassification index (NRI) when baseline hemoglobin concentration was added to the conventional GRACE score [27]. All statistical analyses were conducted using the Stata 14.0 statistical package (Stata Corp, College Station, TX). Statistical significance was tested at a type I error rate of 0.05.

## Results

This study included 200 consecutive ACS admissions to the study center. A total of 28 patients developed MACE only while another 31 died. The observed primary causes of death were: cardiogenic shock (21), septicemia (3), ventricular tachycardia (2), acute left ventricular failure (1), cerebrovascular episode (1), aspiration pneumonia (1), congestive cardiac failure (1) and hypoxic brain injury (1). The study participants who developed either MACE only or died within six months of admission were more likely to be older than 60 years, females and belonging to Killip class III or IV (Table 1). A large proportion of the patients (69%) had ST-elevation myocardial infarction (STEMI). While diabetes (70.5%), hypertension (43%) and cardiovascular disease (ICD-9-CM Diagnosis Code 429.2, 26.6%) were the most common comorbidities, the rarer comorbidities of heart failure and TIA/CVE were more frequent in patients with adverse events (Table 1). The most striking difference between event-free patients and those with adverse events was observed for hemoglobin concentration – either treated as a continuous variable (p = 1.90 × 10^–17^) or as a dichotomous variable indicating anemia (p = 6.72 × 10^–9^). Use of cardiovascular medications showed a generally lower risk of adverse events but was statistically significant for β-blockers and ACE inhibitors only. PCI was done in a total of 18 (9.0%) patients and was not statistically significantly distributed across study outcomes.

**Table 1:** Bivariable association of the baseline characteristics of study patients with the outcomes.

Characteristic	Outcome at the end of six months			P value

	No event (n = 141)	MACE only (n = 28)	Death/MACE (n = 31	

Age [Mean (SD)], y	54.63 (12.31)	62.46 (14.21)	61.39 (13.12)	0.0013^a^
Males [n (%)]	109 (77.30)	13 (46.43)	16 (51.61)	0.0004^b^
Body mass index [Mean (SD)], Kg/m^2^	22.39 (2.11)	22.09 (2.30)	22.17 (2.22)	0.6825^a^
Waist/Hip Ratio [Mean (SD)]	0.91 (0.05)	0.90 (0.05)	0.91 (0.05)	0.7296^a^
Overweight—BMI ≥25 Kg/m^2^ [n (%)]	15 (10.64)	3 (10.71)	4 (12.90)	0.9369^c^
Number of Personal Risk factors [n (%)]^d^				0.4696^c^
0	65 (46.10)	19 (67.86)	17 (54.84)	
1	42 (29.79)	5 (17.86)	7 (22.58)	
2	21 (14.89)	3 (10.71)	6 (19.35)	
3	13 (9.22)	1 (3.57)	1 (3.23)	
Killip class [n (%)]				3.70 × 10^–14c^
I	76 (53.90)	8 (28.57)	2 (6.45)	
II	61 (43.26)	11 (39.29)	11 (35.48)	
III	4 (2.84)	9 (32.14)	15 (48.39)	
IV	0 (0.00)	0 (0.00)	3 (9.68)	
Acute coronary syndrome type [n (%)]^e^				0.3608
STEMI	92 (62.25)	20 (71.43)	26 (83.87)	
NSTEMI	6 (4.26)	1 (3.57)	1 (3.23)	
UA	43 (30.50)	7 (25.00)	4 (12.90)	
Comorbidities [n (%)]				
Diabetes	106 (75.18)	17 (60.71)	18 (58.06)	0.0790^b^
Hypertension	86 (60.99)	13 (46.43)	15 (48.39)	0.2089^b^
TIA/Stroke	0 (0.00)	2 (7.14)	3 (9.68)	0.0020^c^
Heart failure	1 (0.71)	3 (10.71)	3 (9.68)	0.0041^c^
Cardiovascular disease	41 (29.08)	4 (14.29)	6 (19.35)	0.1809^b^
Clinical chemistry at baseline				
Hemoglobin [Mean (SD)], g/dl	12.94 (1.89)	10.16 (1.70)	9.77 (2.59)	1.90 × 10^–17a^
Anemia [n (%)]	63 (44.68)	26 (92.86)	28 (90.32)	6.72 × 10^–9b^
Serum albumin [Mean (SD)], g/dl	4.38 (0.41)	4.51 (0.45)	4.50 (0.44)	0.1411^a^
Serum creatinine [Mean (SD)], mg/dl	1.23 (0.18)	1.27 (0.17)	1.25 (0.22)	0.5892^a^
Serum creatine-kinase MB [Mean (SD)], IU	69.11 (8.07)	68.46 (13.41)	72.97 (10.32)	0.9731^a^
Medication use [n (%)]				
Aspirin	121 (85.82)	20 (71.43)	23 (74.19)	0.0911^b^
β-blockers	105 (74.47)	15 (53.57)	19 (61.29)	0.0489^b^
Statins	110 (78.01)	21 (75.00)	18 (58.06)	0.0697^b^
Angiotensin receptor blockers	16 (11.35)	2 (7.14)	0 (0.00)	0.1256^c^
ACE inhibitors	53 (37.59)	7 (25.00)	4 (12.90)	0.0163^c^
Spironolactone	6 (4.26)	0 (0.00)	1 (3.23)	0.8357^c^
PCI done	14 (9.93)	3 (10.71)	1 (3.23)	0.4663^c^

^a^ estimated using analysis of variance; ^b^ estimated using Pearson’s Χ^2^ test; ^c^ estimated using Fisher’s exact test; ^d^ combinations of tobacco ingestion, smoking and alcohol intake currently or in the past; ^e^ STEMI – ST elevation myocardial infarction, NSTEMI – non-ST elevation myocardial infarction, UA – unstable angina.

Considering these observed bivariable associations shown in Table 1, multivariable logistic regression analyses were performed. In the full model (Table 2), Killip class, history of TIA/Stroke, baseline hemoglobin level and statin use were significantly associated with death within 6 months. When we ran similar analyses for the outcome of death/MACE within 6 months, we found that only Killip class and baseline hemoglobin were significantly associated with this outcome. Together, these results indicated a significant and independent association of baseline hemoglobin levels with both the adverse outcomes.

**Table 2:** Results of logistic regression analysis for the multivariable association of risk factors with the study outcomes.

Covariate	Death within 6 months		Death/MACE within 6 months	

	OR (95% CI)	p	OR (95% CI)	p

**Full model**				

Age	0.98 (0.94–1.02)	0.376	0.99 (0.96–1.03)	0.745
Males	0.92 (0.32–2.62)	0.869	0.74 (0.28–1.89)	0.527
Killip class	11.0 (3.11–38.9)	2.0 × 10^–4^	10.2 (2.54–40.8)	0.001
Diabetes	1.18 (0.41–3.44)	0.755	1.61 (0.64–4.06)	0.307
TIA/Stroke	9.55 (1.01–90.17)	0.049	–	0.992
Heart failure	0.93 (0.14–6.15)	0.940	1.59 (0.14–18.1)	0.709
Hemoglobin	0.70 (0.54–0.91)	0.007	0.58 (0.44–0.75)	4.9 × 10^–5^
Aspirin use	3.73 (0.43–32.1)	0.230	0.69 (0.11–4.56)	0.702
β-blocker use	1.56 (0.37–6.66)	0.546	0.85 (0.25–2.82)	0.792
Statin use	0.23 (0.05–0.98)	0.046	1.10 (0.26–4.66)	0.893
ACE inhibitor use	0.39 (0.10–1.47)	0.165	0.67 (0.24–1.86)	0.444
**Stratified propensity score adjusted model**				

Hemoglobin	0.72 (0.57–0.91)	0.007	0.55 (0.43–0.72)	5.4 × 10^–6^

OR, odds ratio; CI, confidence interval.

To determine the robustness of these observations, we conducted propensity score analyses. Estimation of the propensity score permitted generation of a propensity score based on variables that were balanced across the anemia-driven subgroups (Figure 2A). This propensity score was approximately normally distributed in those who did or did not have baseline anemia (Figure 2B). The propensity score was a significant determinant of both the study outcomes. The significance value for association of the propensity score with death within 6 months and death/MACE within six months was 0.0601 and 0.0041, respectively (data not shown). When quintiles of the propensity score were included in addition to hemoglobin concentration as a covariate, we observed that baseline hemoglobin concentration was highly significantly (p = 0.007) associated with death within 6 months and death/MACE within 6 months (p = 5.4 × 10^–6^; Table 2, propensity score adjusted model). These results further affirmed the independent association of baseline hemoglobin concentration with both the study outcomes.

**Figure 1:**
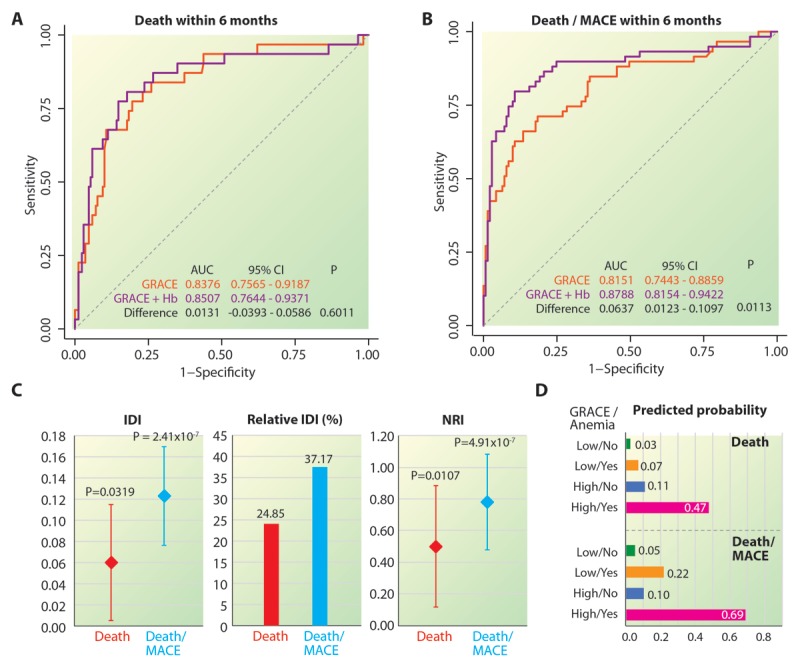
**Incremental value of baseline hemoglobin concentration in prognostication of ACS over the GRACE score. (A–B)** Comparison of the ROC curves that predict death within 6 months (A) or death/MACE within 6 months based on GRACE score only (orange curves) or GRACE score as well as baseline hemoglobin concentration (purple curves). Difference in the areas under the two ROCs were estimated using a bootstrapping method as detailed in the Stata package comproc. Statistical significance was tested using the DeLong, DeLong and Clarke-Pearson test. AUC, area under the curve; ROC, receiver operating characteristic curve. **(C)** Incremental value of baseline hemoglobin concentration as assessed using the incremental discrimination improvement (IDI), relative IDI and the net reclassification index (NRI). Diamonds indicate point estimates and the error bars indicate 95% confidence intervals. All the plots are color-coded for the two study outcomes. **(D)** Predicted probability of the study outcomes based on a combination of the dichotomized GRACE score and presence or absence of baseline anemia.

**Figure 2:**
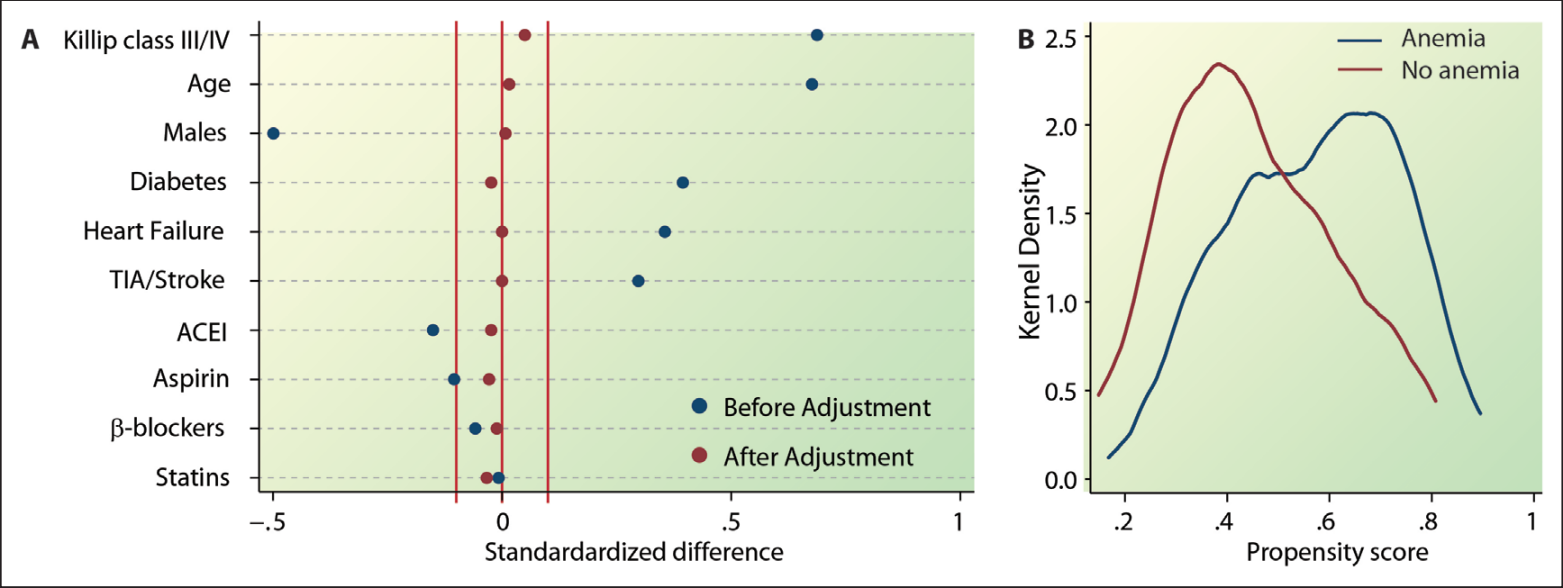
**Derivation of a propensity score to test the robustness of association of baseline hemoglobin concentration with study outcomes. (A)** Sequential logistic regression models were used to ensure the balance of the predictors of baseline anemia across groups of patients with and without anemia. The standardized differences before and after adjustment are shown in blue and red circles, respectively. All the variables were adequately balanced after adjustment. **(B)** Kernel density of the propensity score across patients with and without anemia.

We then determined the incremental value of serum hemoglobin levels over and beyond the GRACE scores for the two outcomes. For death within 6 months, we observed a statistically nonsignificant improvement in the area under the ROC curve (Figure 1A) by addition of baseline hemoglobin levels (p = 0.6011), but there was a clear and significant improvement in the IDI, relative IDI (6%, p = 0.03) and NRI (0.50, p = 0.01) as shown in Figure 1C (red colored diamonds and bars). For the outcome of death/MACE within 6 months however there was a statistically significant improvement in the area under the ROC curve (Figure 1B), as well as IDI (12%, p << 0.0001) and NRI (0.78, p << 0.0001) as depicted in Figure 1C (blue colored diamonds and bars). These observations demonstrated a significant and independent improvement in the prediction of outcomes after ACS when baseline hemoglobin levels were added to the respective GRACE scores of the two outcomes.

From the perspective of clinical utility, we then aimed to stratify the study population into four exclusive groups based on a combination of the GRACE score and presence of anemia. The GRACE score OOPs for the outcomes of death and death/MACE within six months were 113.65 and 149.8, respectively. At these cutoff values, the sensitivity and specificity of the GRACE score to predict death within 6 months was 77.42% and 80.47%, respectively. Similarly, the sensitivity and specificity of the GRACE score to predict death/MACE within six months was 71.19% and 81.56%, respectively. Addition of anemia to the dichotomized GRACE score values yielded the four risk groups shown in Figure 1D. As expected, we observed that for both the outcomes, patients with a high GRACE score and presence of anemia were at a very high risk of developing the outcome. The observed probability of death and death/MACE within six months was 47% and 69% in this risk group, respectively.

## Discussion

Risk stratification and appropriate risk mitigation strategies are of critical importance to reduce the adverse outcomes after ACS [28]. To that end, the GRACE score remains the most popular method of risk stratification of ACS patients. A distinct advantage of the GRACE score is its generalizability since it is based on data from several countries around the world. However, the GRACE Registry data have little representation of rural communities with high prevalence of anemia. Thus, the applicability of the GRACE score in such settings is not well-established. Our results demonstrate that in rural settings where the prevalence of anemia is likely to be high, the prognostic performance of GRACE score can be significantly improved by additively considering the presence of anemia at baseline—a simple clinical measure that can be easily implemented even in the rural settings. It is notable that very few studies [29,30] have attempted to extend the use of the GRACE score to rural settings, and those that have found a need to re-calibrate the GRACE score for such populations.

Our results concur with a burgeoning evidence that baseline hemoglobin measurements improve the prognostic ability of the GRACE score with regards to in-hospital and 30-day mortality [31,32,33], death/MI within 6 months [34,35] or MACEs within 12 months [36]. None of these studies, however, are from a rural setting and have reported prevalence of baseline anemia to be <30%. To our knowledge, our study is the first one to report outcomes of ACS from a rural setting with a substantially higher prevalence of anemia (58%) at baseline. It is noteworthy that the prevalence of anemia reported here is in concordance with other Indian studies having similar patient profiles [7,8]. Together, there now appears to be sufficient evidence to favor the inclusion of baseline anemia in a modified GRACE score for an improved risk stratification of ACS patients.

These findings are important in the light of the continued high prevalence of anemia, especially in low income countries. A large systematic review by Stevens et al. [37], based on 232 nationally representative surveys from 107 countries and 1.9 million hemoglobin measurements, clearly indicates the sustained high prevalence of anemia in Asian and African populations. In the context of acute coronary syndrome, Stucchi et al. [17] reviewed data from 9 published studies (a total of 110,616 patients) across the world, and observed that the prevalence of baseline anemia in ACS patients varied from 10%–43%. In comparison, the baseline prevalence of anemia in this study was 58.5%, in line with the expectation of a higher prevalence in India. In the context of such high prevalence of baseline anemia and its observed independent association with adverse outcomes, inclusion of anemia in a refined GRACE score deserves a closer look.

A limitation of our study is the relatively small sample size. We therefore determined the post hoc statistical power of our study to demonstrate improved prediction of the study outcomes by comparing the area under the ROC curve. Using the R package pROC [38], we found that to detect a significantly improved area under the ROC curve, for the outcome of death within 6 months, our study had a post-hoc power of only 21.40%; but for the outcome of death or MACE within 6 months, our study had a post hoc power of 71.97%. We anticipate more specificity of association with death or MACE rather than death alone (which can be a mixture of various causes of death). Moreover, while the area under the ROC curve demonstrated a moderate increase, both IDI and NRI estimates were strongly and significantly improved by inclusion of baseline anemia with the GRACE score. We thus believe that our study has adequate power to demonstrate the independent and additive association of baseline anemia with the study outcomes.

## Conclusion

Our study points at a need to include baseline anemia as an additional component of the existing GRACE scoring system for prognostication of ACS. This may be especially important in settings where the prevalence of anemia is high. Future studies need to devise and validate the prognostic use of a modified GRACE score that includes baseline anemia.

## Figures and Tables

**Table:** 

ACS	acute coronary syndrome
GRACE	Global Registry of Acute Coronary Events
IDI	incremental discrimination improvement
LMIC	low and middle income countries
MACE	major adverse cardiac event
NRI	net reclassification index
OOP	optimum operating point
ROC	receiver operating characteristics
